# Terthiophene on Au(111): A scanning tunneling microscopy and spectroscopy study

**DOI:** 10.3762/bjnano.2.60

**Published:** 2011-09-09

**Authors:** Berndt Koslowski, Anna Tschetschetkin, Norbert Maurer, Elena Mena-Osteritz, Peter Bäuerle, Paul Ziemann

**Affiliations:** 1 Institut für Festkörperphysik, Albert-Einstein-Allee 11, 89081 Ulm, Germany; 2 Institut für Organische Chemie II und Neue Materialien, Albert-Einstein-Allee 11, 89081 Ulm, Germany

**Keywords:** Au(111), electronic density of states, STM, STS, terthiophene

## Abstract

Terthiophene (3T) molecules adsorbed on herringbone (HB) reconstructed Au(111) surfaces in the low coverage regime were investigated by means of low-temperature scanning tunneling microscopy (STM) and spectroscopy (STS) under ultra-high vacuum conditions. The 3T molecules adsorb preferentially in fcc regions of the HB reconstruction with their longer axis oriented perpendicular to the soliton walls of the HB and at maximum mutual separation. The latter observation points to a repulsive interaction between molecules probably due to parallel electrical dipoles formed during adsorption. Constant-separation (*I*-*V*) and constant-current (*z*-*V*) STS clearly reveal the highest occupied (HOMO) and lowest unoccupied (LUMO) molecular orbitals, which are found at −1.2 eV and +2.3 eV, respectively. The HOMO–LUMO gap corresponds to that of a free molecule, indicating a rather weak interaction between 3T and Au(111). According to conductivity maps, the HOMO and LUMO are inhomogeneously distributed over the adsorbed 3T, with the HOMO being located at the ends of the linear molecule, and the LUMO symmetrically with respect to the longer axis of the molecule at the center of its flanks. Analysis of spectroscopic data reveals details of the contrast mechanism of 3T/Au(111) in STM. For that, the Shockley-like surface state of Au(111) plays an essential role and appears shifted outwards from the surface in the presence of the molecule. As a consequence, the molecule can be imaged even at a tunneling bias within its HOMO–LUMO gap. A more quantitative analysis of this detail resolves a previous discrepancy between the fairly small apparent STM height of 3T molecules (1.4–2.0 nm, depending on tunneling bias) and a corresponding larger value of 3.5 nm based on X-ray standing wave analysis. An additionally observed linear decrease of the differential tunneling barrier at positive bias when determined on top of a 3T molecule is compared to the bias independent barrier obtained on bare Au(111) surfaces. This striking difference of the barrier behavior with and without adsorbed molecules is interpreted as indicating an adsorption-induced dimensionality transition of the involved tunneling processes.

## Introduction

Because of their expedient properties and their diversity, oligo- and polythiophenes are among the most investigated organic semiconductors. Especially, oligothiophenes are very promising candidates for molecular electronics and have been exploited to form organic field-effect transistors [1–2], optical switches [3], light emitting diodes [4], and solar cells [5–7]. To optimize the performance of such devices, the properties of the interface between the organic component and a metal electrode are of utmost importance. Essential parameters, such as the injection characteristics of charge carriers into the organic semiconductor or the temporal stability of a device function, critically depend on this interface behavior. As a consequence, the information on detailed adsorption geometries of a single molecule and on the morphology of extended molecular structures, as well as insight into the electronic structure of molecules on metal surfaces and their dynamic properties are of fundamental interest. All these basic issues can be addressed by scanning tunneling microscopy (STM) on a single-molecule scale. Additionally, quantum chemical properties of a thiophene molecule acting as a single-molecular wire have been investigated [8]. In that specific work, however, the molecule was just weakly coupled to the metal through a thin insulating layer, whereas strong coupling is desirable when contacting a molecular semiconductor to a metal electrode in order to obtain an efficient injection behavior.

Earlier investigations of thiophenes with STM were carried out on self-assembled monolayers (SAMs) mostly under ambient conditions or in the liquid environment of an electrochemical cell [9–11]. In the present contribution we analyze terthiophene (2,2':5',2"-terthiophene), 3T, as a representative of linear oligothiophenes being adsorbed on a Au(111) electrode within the low coverage regime (<1 monolayer (ML)), allowing us to study the interaction of molecules with the substrate on a single-molecule level. Specifically, the topographic and morphological properties of the molecules as well as of their arrays were studied by STM and the corresponding electronic properties by scanning tunneling spectroscopy (STS), both at low temperature to obtain the highest resolution possible. The results reveal many similarities to the corresponding experimental data obtained for 4-mercaptopyridine (4MPy) on Au(111) [12]. This suggests a common STM contrast mechanism for such molecules, at least on this surface. Finally, we discuss the differential barrier height. This not so commonly determined characteristic, describing the voltage dependent tunneling barrier, has been introduced recently in order to remove features of the tunneling tip from STS spectra, assuming the validity of the Wentzel–Kramers–Brillouin (WKB) approximation. Again, 3T and 4MPy on Au(111) exhibit a similar bias dependence of the barrier, which may be attributed to a different dimensionality of the tunneling process on the 2-dimensional surface state and on the localized state induced by the molecule.

## Experimental

Commercially available gold films (typical thickness 250 nm, Arrandee, Germany) on glass were flame annealed in a butane flame to develop extended (111) facets. After introduction into ultra-high vacuum (UHV), these films were further annealed at temperatures up to 700 °C to remove contaminants from the surface. After a routine check of the gold surface by STM at low temperature (*p* < 1 × 10^−10^ mbar, *T* ≈ 5.5 K) [13], the samples were quickly transferred to the preparation chamber under UHV conditions and, there, 0.2 to 0.7 monolayers (ML) of 3T (obtained from Sigma Aldrich and further purified) were deposited onto the gold film at a rate of about 0.01 nm/s from a home-built Knudsen-type cell. After deposition, the samples were immediately transferred back to the low-temperature STM. During the complete transfer and deposition process, the sample holder was kept well below room temperature. A rough estimate from the cooling curve gives *T*_sample_ < −40 °C at any time with molecules on the surface.

For tunneling, W tips were prepared by electrochemically etching of W wires and subsequent annealing of the tips at ~2000 °C in UHV. Finally, tips were conditioned by field emission and desorption at ~1 µA and <1000 V until they showed the expected topographic and spectroscopic STM/STS behavior on Au(111) and Nb(110).

## Results and Discussion

**Figure 1:**
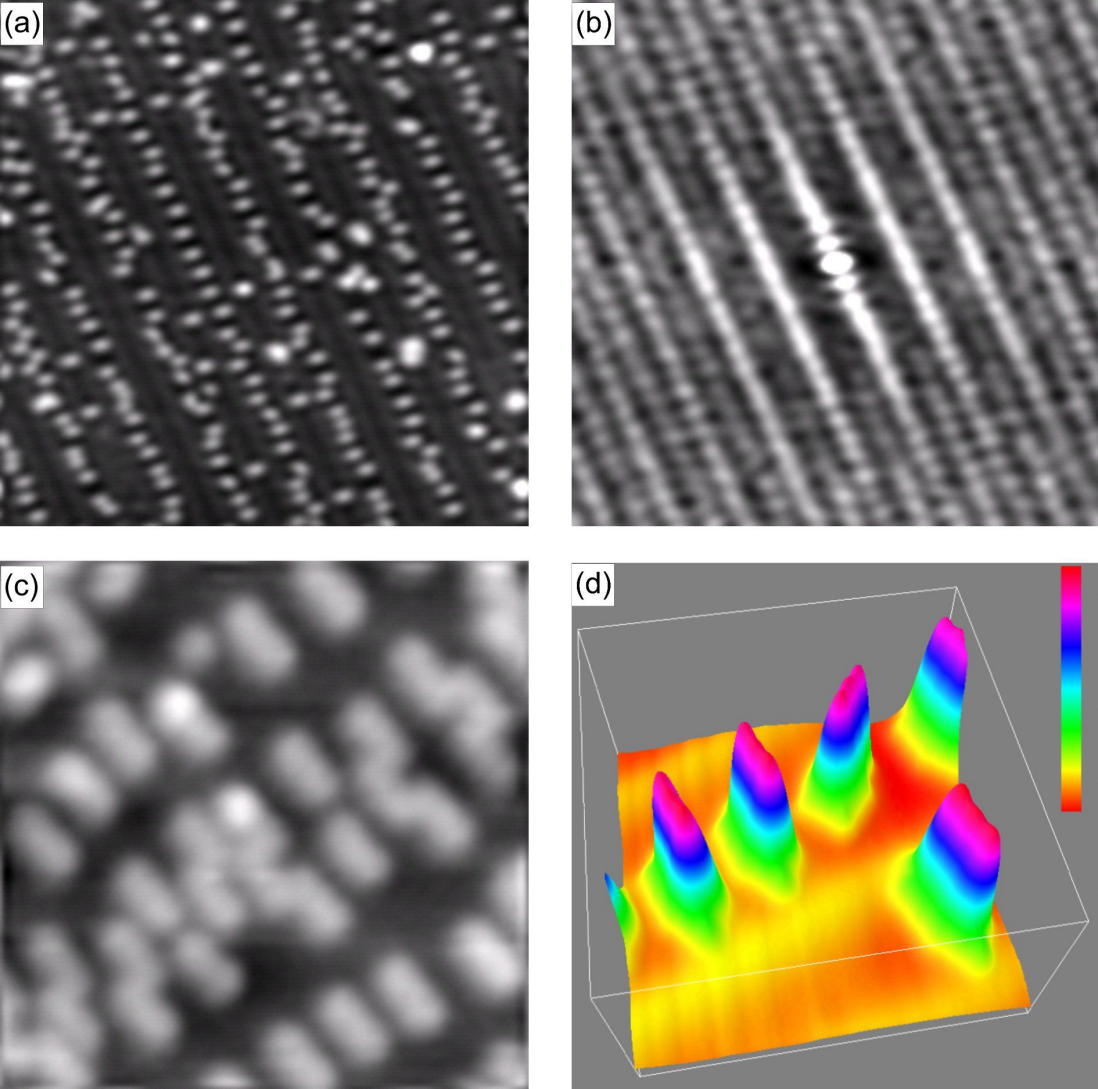
(a) Topographic image of ~0.2 ML of 3T on Au(111) (50 × 50 × 0.17 nm^3^; *I*_set_ = 90 pA, *V*_t_ = −2.0 V); (b) autocorrelation of (a) indicating the abandonment of the hcp regions as well as the equidistant arrangement of the molecules; (c) topographic image of 3T/Au(111) at a coverage of 0.5 ML (7.7 × 7.7 × 0.2 nm^3^; *I*_set_ = 70 pA, *V*_t_ = −1.5 V): 3T form armchair units made from 3 molecules in fcc regions; (d) close-up of a few 3T molecules in 3D representation (7.0 × 7.0 × 0.17 nm^3^; *I*_set_ = 70 pA, *V*_t_ = −1.4 V). The molecules in the upper row reside in an fcc region.

Figure 1(a) shows a topographic image of about 0.2 ML of 3T on Au(111) several days after preparation. One recognizes the elongated molecules on top of a faint corrugation (~10 pm) given by the herringbone reconstruction (HB) of the gold surface (Au(111)-(22 × √3)). Presumably, due to the boundary conditions and strain in the Au film, the HB is straight and the length of the unit mesh may vary from 18 to 22. Whereas immediately after their deposition the 3T molecules are almost randomly distributed on the Au(111) surface, the distribution in Figure 1(a) clearly reveals that the HB serves as a template where (i) the 3T prefer the fcc regions (~93%); (ii) the 3T orient themselves perpendicular to the soliton walls of the HB and, at a lower probability, in multiples of 120° (<
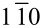
> directions); and (iii) for a given coverage, the 3T apparently maximize their intermolecular distance (here: 2.4 nm). The observed preferential occupation of fcc areas within the HB reconstruction by the 3T translates into a higher binding energy of the molecules at these locations as compared to hcp areas. This is, at least qualitatively, corroborated by the fact that molecules from within hcp areas can be easily made to move by scanning the STM tip, or even made to jump onto the tip, whereas corresponding movements of molecules from fcc areas demand tunneling currents considerably higher than 100 pA and also a tunneling bias, *V*_t_, close to the lowest unoccupied molecular orbital (LUMO) of 3T.

A preferential adsorption of the aromatic molecules in the fcc regions has also been observed for azobenzene [14], 1-nitronaphthalene [15–16], tetrathiafulvalene [17], and 9-aminoanthracene [18]. The tendency of the molecules to maximize the intermolecular distances may point to some charge transfer between molecule and substrate upon adsorption [17]; the resulting dipoles would be perpendicular to the surface and parallel to each other, and thus a repulsive interaction is expected. Both, the preferential occupation of fcc areas by the molecules as well as their equidistant chain-like arrangement are made even more clearly visible by the autocorrelation function of Figure 1(a) as presented in panel (b).

At higher coverage, 3T adsorbs increasingly in hcp regions. Concurrently, 3T aggregates are formed in fcc regions. These small aggregates are armchair structures which are perpendicularly aligned with the HB as seen in the center of Figure 1(c). These 3D aggregates, however, are not stable under STM conditions and their formation is in contrast to the observation of long range ordering of α-sexithiophene on Au(111) [19]. A possible reason could be the considerably weaker interaction of 3T with the substrate due to its shorter chain length.

**Figure 2:**
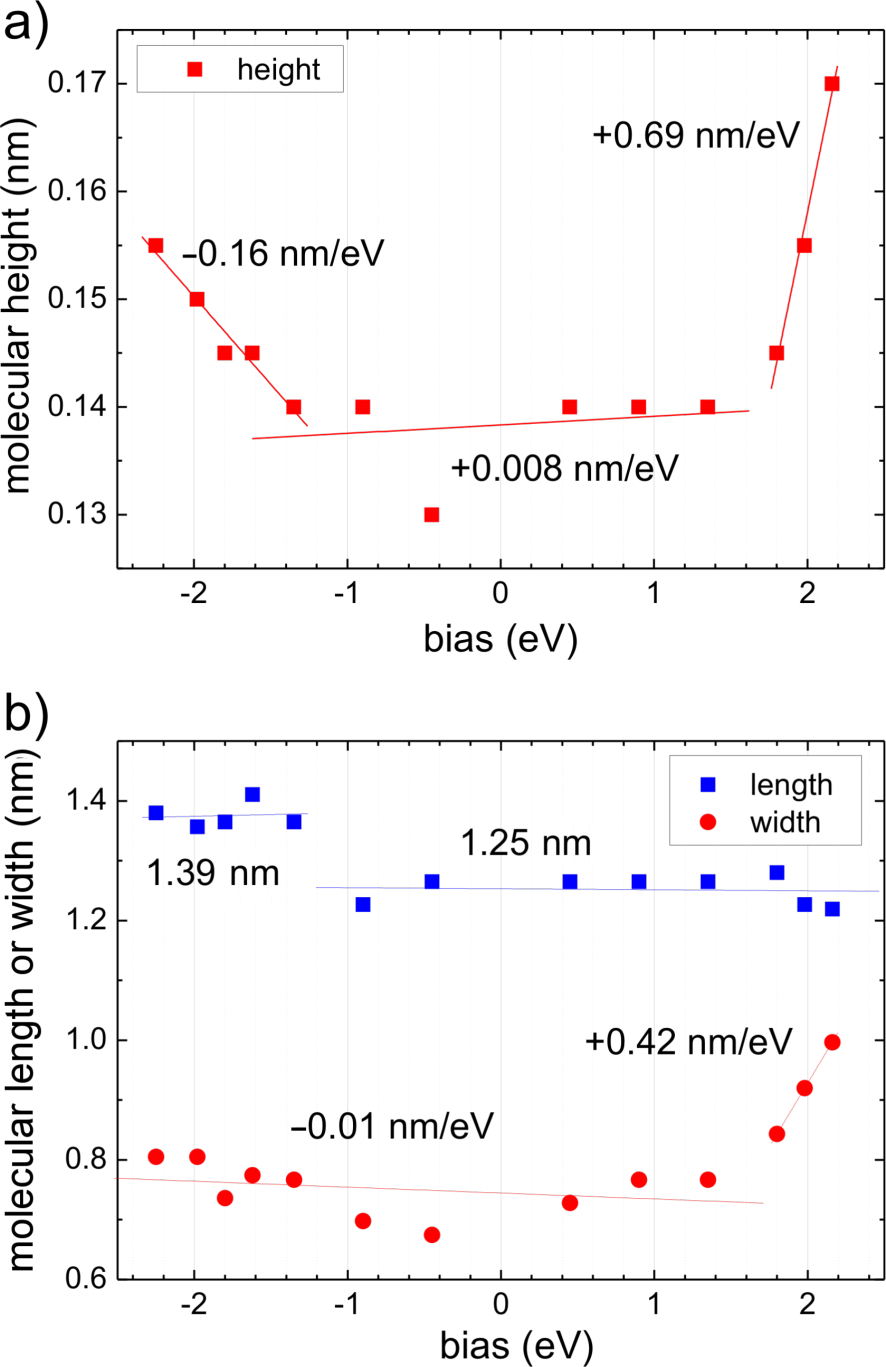
Apparent (a) molecular height, *h*, and (b) molecular length, *l*, and width, *w*, of 3T/Au(111). Solid straight lines indicate linear fits to sections of data discussed in the text; slopes/constant values are assigned to each.

The appearance of 3T/Au(111) is bias dependent in STM. In Figure 1 all molecules appear as elongated entities. In a close-up, Figure 1(d), one recognizes two faint constrictions (3–5 pm) in the molecules at the positions of the two bonds between the thiophene rings. At higher bias, the molecules appear more rounded in STM and become almost spherical at a bias of *V*_t_ > +2.0 eV. Figure 2(a) and Figure 2(b) summarize the bias dependent morphology of 3T. The molecular height is almost independent of bias, *V*_t_, when │*V*_t_│ < 1.5 eV, with a value of 0.14 nm. For lower and higher values of *V*_t_ (< −1.5 eV or > 1.5 eV) the molecular height increases linearly although with different slopes (−0.16 nm/eV and 0.69 nm/eV in the two regimes given above, respectively). The length of the molecules is almost constant for *V*_t_ < −1.2 eV at 1.39 ± 0.07 nm and for *V*_t_ > −1.2 eV at 1.25 ± 0.01 nm (Figure 2(b)). Finally, also the molecular width is almost constant for *V*_t_ < 1.5 eV at 0.7 nm and increases to approximately the molecular length at *V*_t_ ≈ 2.4 eV. At low bias the size of 3T is slightly larger as compared to the geometric size of a free 3T molecule (1.26 nm × 0.47 nm), which can easily be explained by the finite radius of curvature of the tunneling tip. The height, however, is considerably smaller than expected. Kilian et al. measured the height of quaterthiophene on Ag(111) by X-ray standing waves and determined a separation of the molecules from the surface of 0.315 nm [20]. Such a considerable difference requires an explanation. Typically one would argue in STM that a change of the density of states (DOS) or the barrier height, Φ, above a molecule changes the tip–sample separation. However, the spatial distribution of the DOS may also change. We will discuss this in more detail later.

**Figure 3:**
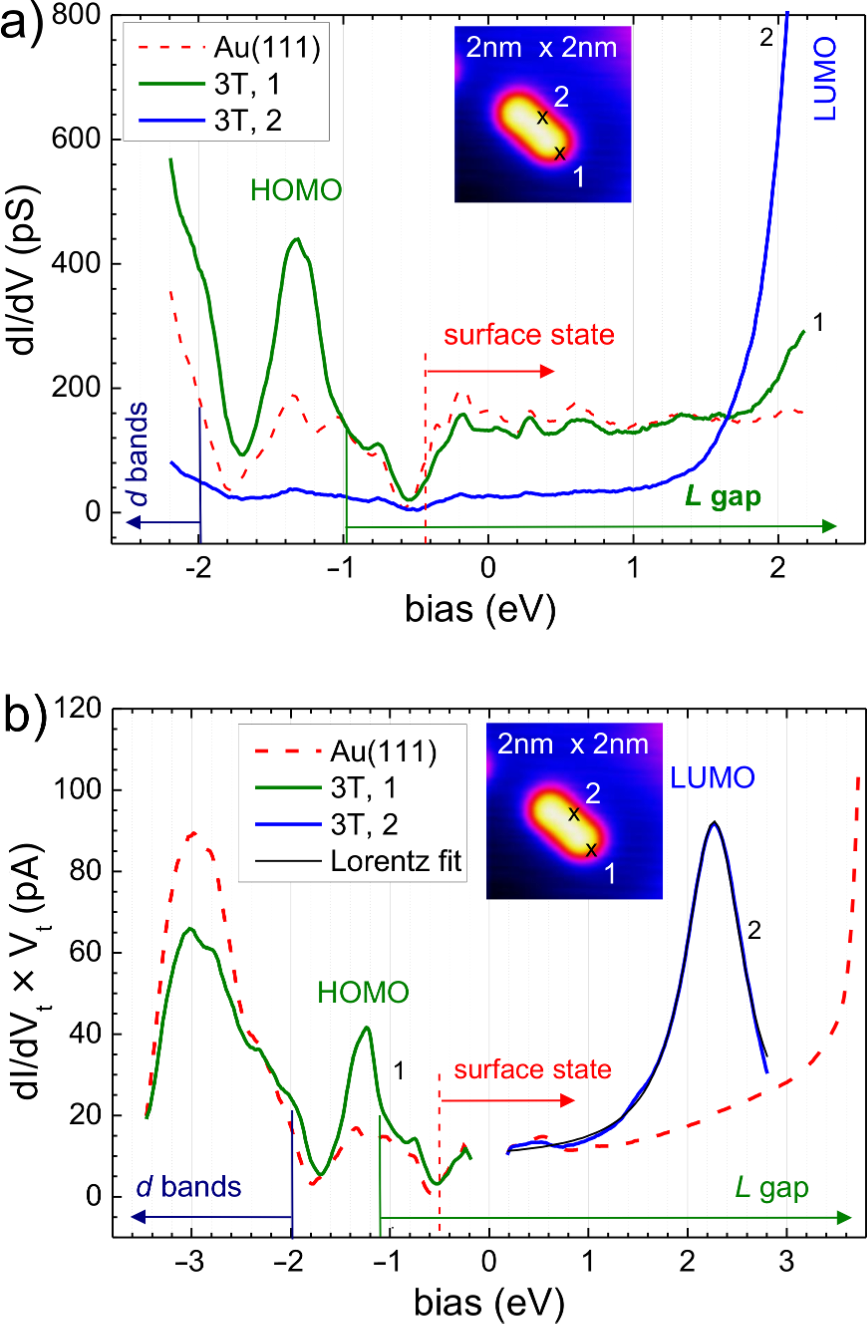
STS of 3T/Au(111): (a) constant-separation (*I*-*V*) spectra taken on the bare gold surface significantly far away from the molecule (red), taken at the center of the flanks (position 2 in the inset) (blue), and taken at an end of the molecule (position 1 in the inset) (green); the initial setpoint before disabling the feedback loop is approximately given by the intersection of the curves at (+1.6 eV, 150 pS); (b) constant-current (*z*-*V*) spectra (*I*_set_ = 29 pA) taken at the bare gold surface (red curves) and in the flanks (blue) or the ends (green) of the molecule to detect the LUMO and the HOMO, respectively. Also shown is a Lorentzian fitted (black curve) to the positive branch on the molecule (center 2.26 eV, width 0.71 eV corresponding to a lifetime of ~0.5 fs).

In order to understand the morphology of the 3T/Au(111) we performed STS. Since 3T is an extended object in STM, one must take into consideration that the STS results may depend on the specific location on a molecule where spectra are taken. Figure 3(a) displays *I*-*V* spectra recorded on the bare Au(111) (dashed red curve), at the end of the molecules (green curve, position 1 as indicated in the inset), and in the center of the molecular flank (blue curve, position 2 as indicated in the inset). The bare Au(111) shows the expected behavior with the Shockley surface state for *E* > −0.5 eV, the lower edge of the *L* gap at *E* ≈ −1 eV, and the *d* bands for *E* < −2 eV. At the end of the molecule, the *I*-*V* curve is very similar to the curve on bare gold except for a pronounced peak of the conductivity at −1.3 eV, which is attributed to the HOMO of 3T/Au(111), and there is a minor but significant increase for *E* > 2 eV caused by the LUMO of 3T/Au(111). In the flanks, the *I*-*V* curve corresponds, to a good approximation, to 1/5 of the *I*-*V* curve measured on bare Au(111) with a pronounced increase for *V*_t_ > 1.0 eV which is due to the LUMO of the molecule.

To resolve the electronic structure in an extended energy range covering more of the *d* bands and the entire LUMO, we performed constant-current (*z*-*V*) spectroscopy, where the tunneling current is kept constant while the tip–sample separation, *z*, and the differential conductivity, ∂_V_*I*, is recorded. As shown previously [12], a more appropriate quantity to compare to the DOS of the sample is the product ∂_V_*I* × *V*_t_ since, when plotting versus *V*_t_, the singularity of ∂_V_*I* at *V*_t_ = 0 is lifted.

Besides the features already mentioned above, these *z*-*V* spectra show additionally the upper edge of the *L* gap on the bare gold at *V*_t_ ≈ +3.7 eV, and, most importantly, one finds a pronounced peak on the molecule, which we attribute to the LUMO of the molecule. In this representation (Figure 3(b)), the LUMO of a molecule in the fcc region of the HB can be nicely fit by a Lorentzian with the center at *V*_t_ = +2.26 eV and a width of 0.71 eV, which corresponds to a lifetime of electrons in the LUMO of ~0.5 fs.

**Figure 4:**
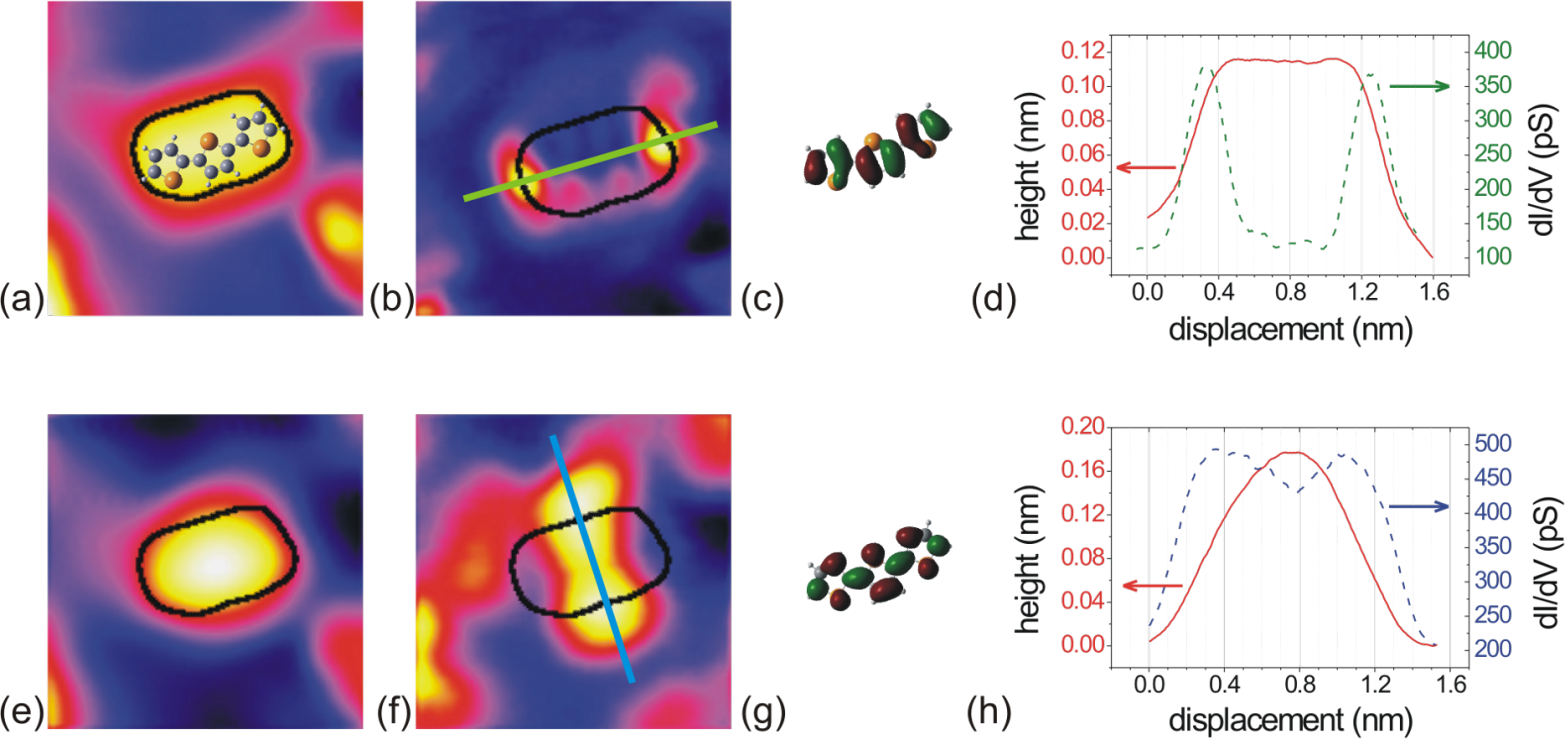
(a) and (e) shape of single 3T molecule on Au(111) (2 × 2 × 0.15) nm^3^, (b) and (f) ∂_V_*I* maps showing the spatial distribution of the electron density of the HOMO (*V*_t_ = −1.14 eV, *I*_t_ = 56 pA for (a) and (b)) and the LUMO (*V*_t_ = 2.28 eV, *I*_t_ = 100pA for (e) and (f)), respectively. (d) and (h) show line profiles across the molecule in the topographies (a) and (e), together with the profile across the ∂_V_*I* maps (b) and (f), respectively. (c) and (g) represent the electron density for 3T of (c) the HOMO and (g) the LUMO, respectively, as calculated by the program “Gaussian03”. Scales and the orientation of the molecule in (c) and (g) are approximately equal to (a) and (e).

The spatial distribution of electronic states at an energy *E* can be imaged by measuring the conductivity of the tunneling junction simultaneously with topography at a given bias *V*_t_ = *E* and a bias modulation at a frequency well above the cut-off frequency of the topographic feedback loop. Figure 4 displays the topography (left column, (a) and (e)) of a single molecule that was scanned twice, once with *V*_t_ = –1.14 eV close to the HOMO (upper row) and once with *V*_t_ = +2.28 eV close to the LUMO (lower row). The corresponding conductivity maps are shown in the second column of Figure 4 for the HOMO (Figure 4(b)) and for the LUMO (Figure 4(f)). The lateral drift is negligible resulting in a excellent reproducibility of the lateral position during consecutive scans. The contour of the topography (half height of the molecule, black line) is drawn in the ∂_V_*I* maps for better orientation and comparison. For clarity, we took also cross sections from topography and conductivity maps along the lines included in the conductivity maps. These profiles are displayed in Figure 4(d) and Figure 4(h). Accordingly, the main maxima of the HOMO are located exactly at the ends of the molecule with four additional minor maxima in between at the boundary of the molecule, two on each side. The LUMO is located exactly at the flanks of the molecule appearing like the wings of a butterfly. Consequently, the molecule appears slightly longer by ~0.14 nm at biases below –1.2 eV (Figure 2(b)), and it gets broader when approaching the bias corresponding to the LUMO, in accordance with the results shown in Figure 2. Relative to the long axis of the molecular contour, the minor maxima of the HOMO are typically very asymmetric while the LUMO appears symmetric relative to the same axis. Thus, the HOMO allows identification of the orientation of the 3T molecules. Comparison to a ball-and-stick model of the 3T molecule, as inserted in Figure 4(a), and to the HOMO–LUMO distributions of a free 3T as calculated by Gaussian03, and given in panels Figure 4(c) and Figure 4(g), respectively, suggests that the observed asymmetry can be attributed to the fact that the central sulfur atom does not contribute significantly to the HOMO of a free molecule, as opposed to its contribution to the LUMO. Thus, with respect to symmetry, the adsorbed 3T molecule shows some resemblance to the free molecule behavior. In closer detail, however, clear indications of the influence of adsorption are visible, such as the butterfly shape of the LUMO.

**Figure 5:**
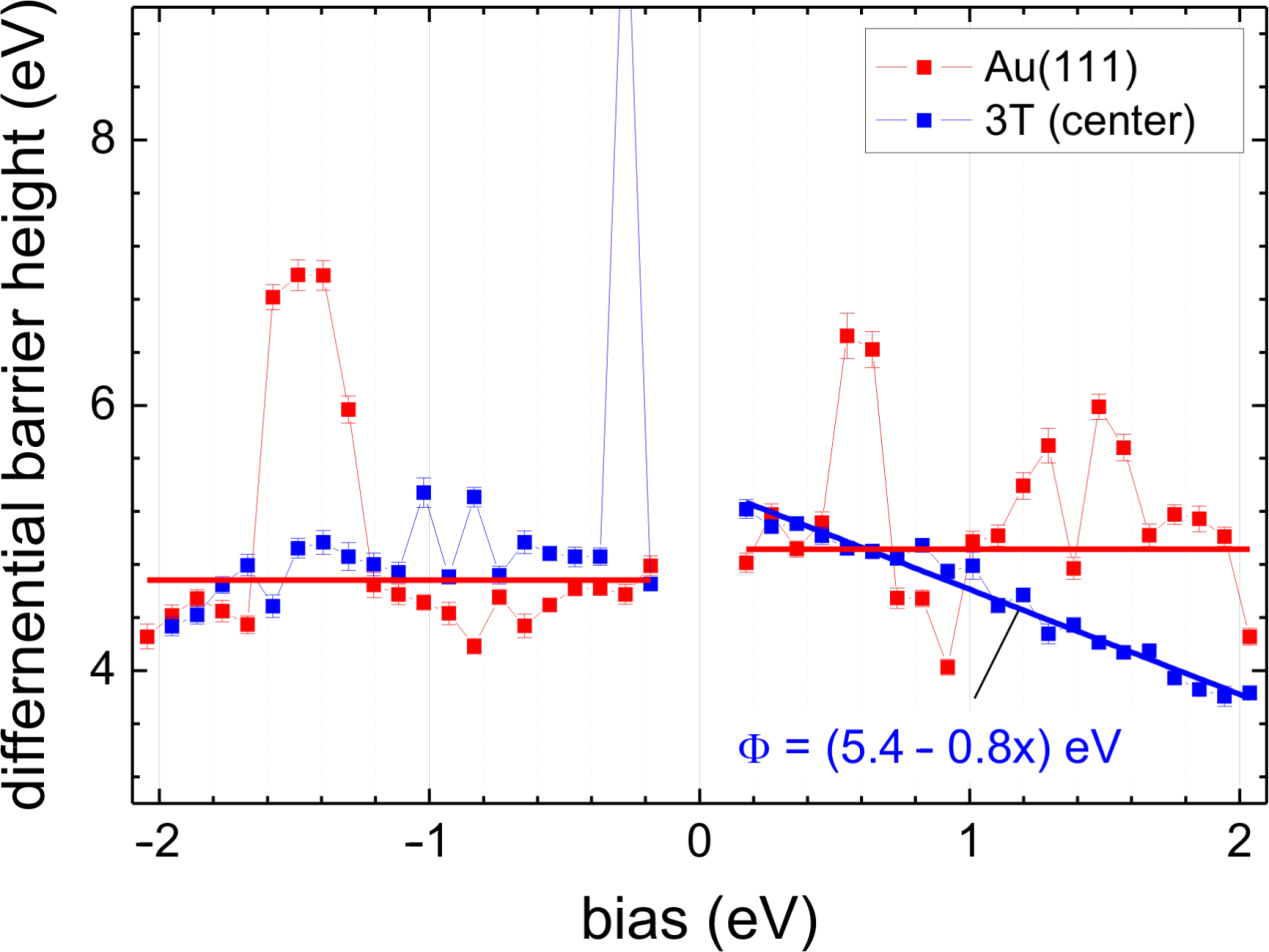
Differential barrier height Φ_diff_ = (∂_z_∂_V_*I*/∂_V_*I*)^2^ on (blue) and off (red) the 3T molecule. The straight lines are linear fits to the positive and negative branches, respectively (red: On Au(111); blue: In the center of 3T). The differential barrier of gold here is almost constant at Φ_diff_ ≈ 4.8 eV. The differential barrier on 3T drops off linearly for positive bias and is slightly greater at small bias compared to bare Au(111).

We return to the discussion of the contrast mechanism of 3T in STM. At low bias, as mentioned above, the 3T molecule appears to have a height of 0.125 nm in STM images while X-ray standing wave measurements suggest a separation of 0.315 nm between the nuclear planes of the gold surface and the molecule. To clarify this significant discrepancy from the STM’s point of view, the differential barrier height defined by Φ_diff_ = (∂_z_∂_V_*I*/∂_V_*I*)^2^ [21] was determined. The differential barrier, Φ_diff_, may be thought of as similar to the commonly used apparent barrier height defined by Φ_app_ = (∂_z_*I*/*I*)^2^, which is bias dependent. Due to differentiation, however, Φ_diff_ is much more sensitive to changes in the tunneling probability at the Fermi levels as compared to Φ_app_. The corresponding results for Φ_diff_ measured on and away from the molecule are displayed in Figure 5. For the bare Au(111), Φ_diff_ is almost constant at Φ_diff_ = 4.8 eV over the complete bias range from −2 eV to +2 eV [22]. Though the absolute value of Φ_diff_ may vary from sample to sample within a range from 4 eV to 5 eV, the constancy of those values independent of the applied bias, *V*_t_, is typical for Au(111) surfaces [12]. On the molecule, Φ_diff_ is slightly higher at low bias and decreases linearly for positive bias. Importantly, the barriers on and away from the molecule are not sufficiently different to change the tip-sample separation significantly in these two positions. Thus, barrier effects cannot be responsible for the above-mentioned height discrepancy. Furthermore, since STS reveals that the electronic properties of 3T/Au(111) are almost identical to those of bare Au(111) (Figure 3) in the bias range −1 eV to +1.5 eV, one concludes that STM performed at low bias on 3T/Au(111) senses a Au surface state that is modified by the molecular adsorption. Assuming, similarly to the case of 4-mercaptopyridine (4MPy) on Au(111) [12], that the final tunneling state is simply the Au(111) surface state shifted outwards from the surface due to the presence of the molecule, this shift can be identified from the experimentally determined height of the 3T molecule at low bias (0.14 nm).

At a bias *V*_t_ > 1.5 eV the LUMO of the molecule contributes perceptibly to the density of states (DOS) and, to maintain the current setpoint under feedback, the tip must be withdrawn from the surface with increasing bias in accordance with the DOS of the LUMO, thus leading to an enhanced apparent height of the 3T molecule. For the LUMO energy, a molecular height of approximately *h*_m_ ≈ 0.2 nm (Figure 2(a)) with respect to the surface state on the bare Au(111) is extrapolated. Combined with the expectation that the position of the undisturbed Au(111) surface state is approximately half a Fermi wavelength in front of the nuclear surface plane (0.15 nm), this adds up to an apparent molecular height, *h*_m_, of about 0.35 nm in excellent agreement with the X-ray standing-wave experiment [20] and, thus, resolving the above discrepancy.

It is interesting to mention here, that, as in the case for 4MPy/Au(111), also 3T/Au(111) exhibits a linear dependence of Φ_diff_ for positive bias with a slope close to −1. This behavior is significantly different from the bias-independent barrier height on the bare Au(111) surface. As mentioned previously [12], such a linear bias dependence of the differential barrier as measured on the molecules is hard to understand within the three-dimensional WKB approximation, since, in that case, the parallel component of the energy *E*_p_ (perpendicular to the tunneling direction *z*) formally adds to the effective barrier (the transmission probability function changes from


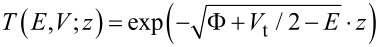


in one dimension to


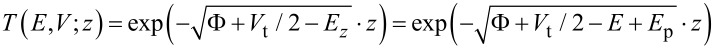


in three dimensions with *E* = *E*_z_ + *E*_p_). At positive bias we may assume, for simplicity, that the states aligned with the upper Fermi level (tip) dominate the barrier measurement. Additionally, for 3T as well as for 4MPy on Au(111), the sample DOS does not influence the barrier measurement since it is constant [21] at least in the low bias range. Consequently, we may set *E* = *V*_t_ and, hence, *E*_p_ ≈ −*E*/3 to *E*_p_ ≈ −*E*/2 to explain the measurement of Φ_diff_. On the other hand, we attributed the involved sample states in that energy range to a modified surface state which means extending the 2D state into the 3^rd^ dimension. We believe that the characteristic change in the behavior of Φ_diff_, from being approximately constant on Au(111) to dropping off almost linearly on a molecule, is a manifestation of that change in dimensionality. However, presently there are still too many open questions as to the role of the tip (dispersion) and even whether there would be principal limits to the WKB approximation.

## Conclusion

Low-temperature scanning tunneling microscopy and spectroscopies (STM, STS) under ultra-high vacuum conditions were applied to investigate the structural and electronic properties of terthiophene molecules (3T) adsorbed on Au(111) in the submonolayer regime. The data clearly revealed that the standard herringbone reconstruction (HB) of the Au(111) surface acts as a template, with 3T molecules preferentially adsorbed on its fcc regions in a perpendicular orientation with respect to the soliton walls of the HB. Adsorbed in this way, the 3T molecules exhibited almost identical interparticle distances pointing to repulsive intermolecular interactions, probably due to electrical dipoles formed by the adsorption. The lateral variation of the adsorption energy appeared to be relatively small allowing for tip-induced rotations and displacements of the molecules, depending on the tunneling current and the bias.

The shape of a single 3T molecule on Au(111) depended merely on the bias for values below +1.5 eV. For a bias above +1.5 eV, the shape of the molecule was strongly influenced by tunneling into its LUMO. The STM height of the molecules was shown to have special significance. Emphasizing here the bias regime between HOMO and LUMO, the STM appearance of the 3T was found to be governed by a modified Shockley-like surface state of Au(111), which is shifted outwards from the gold surface in the presence of the molecule. Additionally, when adding the separation of the Shockley-like surface state from the nuclear plane of the Au surface atoms to the apparent STM height of the 3T molecules on Au(111) measured at a bias corresponding to the LUMO, one obtains a value for the molecule–surface separation close to that which was reported for X-ray standing-wave experiments.

The energetic positions of both, the HOMO and LUMO, could be precisely determined by constant-current STS resulting in values of −1.3 eV and +2.26 eV, respectively. While most data suggest a relatively weak interaction of 3T with the gold surface, the lateral distribution of the LUMO–HOMO is significantly different from the expectation based on calculated electron distributions of a free 3T molecule. While the LUMO is mostly located at the center of the flanks of the molecules, leading to a butterfly-like appearance of this distribution, the HOMO is located at the ends of the molecule and shows a clear asymmetry relative to the axis of the 3T molecules. Combining LUMO–HOMO STS data, thus, allows determination of the orientation of a 3T molecules on Au(111).

Finally, the differential barrier on and away from the 3T/Au(111) was determined and similar results were obtained as previously reported for 4-mercaptopyridine on Au(111). In both cases, the differential barrier shows a linear drop-off at positive bias as opposed to the practically bias-independent behavior on the bare Au(111) surface. Referring to the expected WKB behavior and combining with the presently reported results leads to an interpretation in terms of a dimensional crossover induced by performing STS on top of and through a 3T molecule.
